# Clinical experience with the novel histone deacetylase inhibitor vorinostat (suberoylanilide hydroxamic acid) in patients with relapsed lymphoma

**DOI:** 10.1038/sj.bjc.6603464

**Published:** 2006-12-12

**Authors:** O A O'Connor

**Affiliations:** 1 Laboratory of Experimental Therapeutics, Department of Medicine, Division of Hematologic Oncology, Lymphoma Service, Memorial Sloan Kettering Cancer Center, New York, NY, USA

**Keywords:** histone deacetylase, non-Hodgkin's lymphoma, vorinostat, SAHA, Hodgkin's disease, BCL-6

## Abstract

Preclinical studies indicate that vorinostat (suberoylanilide hydroxamic acid or SAHA) inhibits histone deacetylase (HDAC) activity, increases acetylated histones H2a, H2b, H3, and H4, and thereby induces differentiation and apoptosis in a variety of tumour cell lines, including murine erythroleukaemia, human bladder transitional cell carcinoma, and human breast adenocarcinoma. On the basis of these favourable preclinical findings, vorinostat has been selected as a candidate for clinical development with the potential to treat patients with selected malignances, including Hodgkin's disease and non-Hodgkin's lymphomas. Phase I clinical trials in patients with haematological malignances and solid tumours showed that both intravenous (i.v.) and oral formulations of vorinostat are well tolerated, can inhibit HDAC activity in peripheral blood mononuclear cells and tumour tissue biopsies, and produce objective tumour regression and symptomatic improvement with little clinical toxicity. The dose-limiting toxicities (DLT) of i.v. vorinostat were primarily haematologic and were rapidly reversible within 4–5 days of therapy cessation. In contrast, the DLT for oral vorinostat were primarily non-haematologic (including dehydration, anorexia, diarrhoea, fatigue) and were also rapidly reversible, usually within 3 days. Further research is warranted to optimise the dosing schedule for vorinostat, particularly with respect to dose, timing of administration, and duration of therapy, and to fully delineate the mechanism(s) of antitumour effect of vorinostat in various types of malignances. Several phase II studies are currently ongoing in patients with haematological malignances and solid tumours.

The potential therapeutic benefit created by facilitated gene transcription has led to the development of histone deacetylase (HDAC) inhibitors, which have the capacity to induce cytodifferentiation, and cause cell-cycle arrest and apoptosis of transformed cells (Marks *et al*, 2004). One such HDAC inhibitor, vorinostat (suberoylanilide hydroxamic acid or SAHA), has been shown to induce differentiation of murine erythroleukaemia (Richon *et al*, 1996), human bladder transitional cell carcinoma (T24) (Richon *et al*, 2000), and human breast adenocarcinoma (MCF-7) (Huang and Pardee, 2000; Munster *et al*, 2001). Vorinostat inhibits HDAC through a direct interaction with the enzymes catalytic site, resulting in accumulation of acetylated histones H2a, H2b, H3, and H4. Based on the biological rationale and early preclinical data summarised above, vorinostat was selected as a candidate for clinical development. This article will summarise the toxicity, pharmacokinetic and pharmacodynamic profiles, and efficacy of intravenous (i.v.) and oral formulations of vorinostat, and review the findings from initial phase I studies that revealed interesting differences in toxicity profile between the two formulations. Additional reviews regarding the biological aspects of HDACs can be found elsewhere (Marks *et al*, 1999; Marks *et al*, 2000; Marks *et al*, 2001; Dokmanovic and Marks, 2005; Marks and Dokmanovic, 2005).

## BCL-6 AS A THERAPEUTIC TARGET—RATIONALE FOR USE OF VORINOSTAT TO TREAT LYMPHOMA

**Figure 1:**
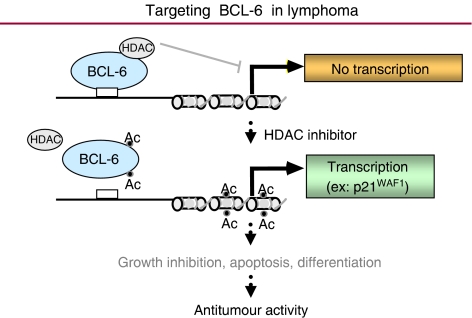
BCL-6 is constitutively expressed in a large proportion of B-cell lymphomas and is involved in the suppression of genes involved in the control of lymphocyte activation, differentiation, and apoptosis. In the acetylated state, BCL-6 is inactive and loses its ability to repress transcription and to induce cell transformation. Pharmacological inhibition of HDAC with agents such as vorinostat may result in the accumulation of acetylated BCL-6, expression of the cell-cycle regulator p21^WAF1^ and ultimately growth inhibition, apoptosis, and differentiation of B-cell lymphoma cells.

The proto-oncogene BCL-6, located at chromosome 3q27, encodes a POZ/zinc finger sequence-specific transcriptional repressor and represents one of three genes commonly implicated in non-Hodgkin's lymphoma (NHL)(the other two genes are BCL-2 and c-myc) (Ye *et al*, 1993a, 1993b; Lo *et al*, 1994; Kramer *et al*, 1998; Pasqualucci *et al*, 2003). BCL-6 is constitutively expressed in a large proportion of B-cell lymphomas where it appears to suppress genes involved in the control of lymphocyte activation, differentiation, and apoptosis (Pasqualucci *et al*, 2003). Recent studies suggest that the transcriptional co-activator p300 acetylates BCL-6 and, in doing so, disrupts the ability of BCL-6 to recruit HDACs, thereby compromising its ability to repress transcription and induce cell transformation (Bereshchenko *et al*, 2002). This has important therapeutic ramifications in that pharmacological inhibition of HDACs might be expected to result in the accumulation of the inactive acetylated BCL-6 which can then induce cell-cycle arrest and death of B-cell lymphoma cells (Figure 1). This hypothesis is supported by the observation that the HDAC inhibitor trichostatin A (TSA) results in apoptosis in several cell lines of B-NHL(Pasqualucci *et al*, 2003). Collectively, these studies suggest that HDAC inhibitors such as vorinostat may represent a novel therapeutic approach to treat patients with certain types of lymphomas.

## INTRAVENOUS AND ORAL FORMULATIONS OF VORINOSTAT

**Figure 2:**
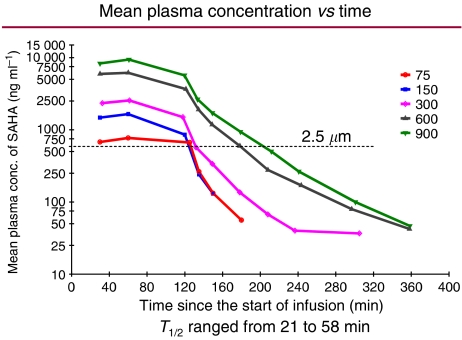
Mean plasma vorinostat concentrations over time following escalating i.v. vorinostat doses in patients with advanced cancer. Reproduced with permission from Kelly *et al* (2003).

**Figure 3:**
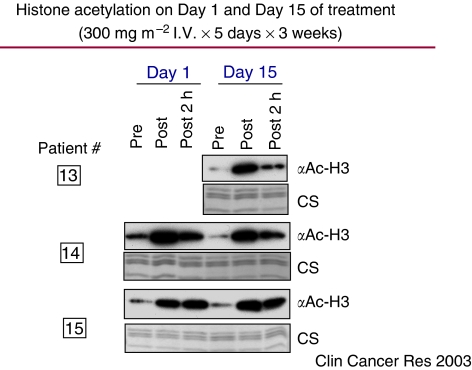
Effect of i.v. vorinostat on histone acetylation in mononuclear cells (by Western blot analysis using a rabbit purified polyclonal anti-acetylated histone H3 antibody) obtained from three cancer patients. As a loading control for histone proteins, parallel gels were run and stained with Coomassie (CS). Peripheral whole blood was collected from each patient before (pre) and at the end of a 2-h vorinostat infusion (post), as well as 2 h after completion of infusion (post 2 h).

Dose-escalating studies with daily 2-h i.v. infusions of vorinostat administered 5 days per week for up to 3 consecutive weeks demonstrated that an i.v. formulation could be administered safely to patients with haematological and solid tumours (Kelly *et al*, 2003). Peak vorinostat concentrations occurred approximately 60 min after beginning the infusion (range 25–120 min). The terminal half-life of i.v. vorinostat ranged from 21 to 58 min, and the area under the plasma concentration curve was proportional to the dose administered. At all i.v. doses of vorinostat studied (75, 150, 300, 600, and 900 mg m^−2^ day^−1^), plasma concentrations exceeded 2.5 *μ*M (Figure 2), a concentration that inhibits cell proliferation *in vitro* and results in accumulation of acetylated histones. In addition, i.v. vorinostat was also shown to inhibit HDAC activity in normal cells (peripheral blood mononuclear cells) as well as in tumour tissue biopsies. As illustrated in three patients, accumulation of acetylated histones in peripheral blood mononuclear cells occurred at the end of a 2-h vorinostat infusion and was still evident 2 h after the infusion ended (Figure 3).

To simplify the administration of vorinostat, an oral formulation was developed, which also shows a favourable pharmacokinetic profile while retaining its antitumour activity. The bioavailability of oral vorinostat is relatively high (43%) and appears to be uninfluenced by the consumption of food (Kelly *et al*, 2005). Oral vorinostat demonstrated linear pharmacokinetics from 200 to 600 mg, although peak concentrations were lower than that seen with the i.v. formulation (Kelly *et al*, 2005). The apparent half-life of oral vorinostat ranged from 91 to 127 min, values that were 2–3-fold higher than those determined after i.v. administration. Plasma concentrations of vorinostat were detectable at least 10 h post-ingestion, whereas vorinostat was undetectable in plasma 4–6 h after i.v. dosing. As observed following i.v. administration, oral vorinostat consistently effected accumulation of acetylated histones in peripheral blood mononuclear cells at 2 h post-dosing, an effect that persisted for up to 10 h after a single dose of 400 mg or higher (Kelly *et al*, 2005). Histone acetylation was still apparent in patients receiving oral vorinostat for 6 months or longer.

## COMPARISON OF ORAL AND I.V. VORINOSTAT IN PHASE I STUDIES

The antitumour effects of oral and i.v. formulations of vorinostat have been assessed in two Phase I clinical trials (Kelly *et al*, 2003, 2005). In both of these trials, escalation of vorinostat was carried out in parallel and independently in patients with solid tumours and haematological malignances. The present review primarily focuses on data obtained in patients with advanced haematological malignances who had failed or relapsed following standard therapy (O'Connor *et al*, 2006). The patient population included those with refractory or relapsed leukaemia, multiple myeloma, indolent or aggressive NHL, mantle cell lymphoma, and Hodgkin's disease (HD). Eligible patients were required to have a Karnofsky performance status of at least 70, adequate renal and hepatic function, an absolute neutrophil count of ⩾500 cells mm^−3^, and a platelet count of >25000 cells mm^−3^, and no serious comorbidities.

A total of 39 patients with haematological malignancy were registered, of which 35 were treated with either i.v. vorinostat or oral vorinostat (Table 1). Compared with the i.v. vorinostat group, patients receiving oral vorinostat showed a greater variation in the types of underlying haematological malignancy. The majority of patients in both groups were heavily pretreated, with some patients having received up to 15 different chemotherapy or biological therapies before study enrolment. In addition, between 35 and 50% of patients had received prior stem cell transplantation and approximately half of all patients had previously received therapy with rituximab. The duration of therapy was longer in patients receiving oral vorinostat compared with patients receiving i.v. vorinostat (average 14.1 *vs* 10.5 weeks, respectively). However, the largest difference between the study groups was age, with patients receiving i.v. vorinostat being considerably younger than patients receiving oral vorinostat (median age 39 *vs* 57 years, respectively).

## SAFETY AND TOLERABILITY

Overall, both vorinostat formulations were well tolerated (Kelly *et al*, 2003, 2005; O'Connor *et al*, 2006). Major adverse events with oral vorinostat included fatigue, diarrhoea, anorexia, and dehydration, whereas myelosuppression and thrombocytopenia were more prominent with i.v. vorinostat (Table 2). Typically, the haematological toxicities, especially the thrombocytopenia, resolved shortly after vorinostat therapy was stopped, suggesting that toxicity did not affect megakaryocytes directly, but may have add an affect on the terminal budding stage of platelet formation. This was confirmed on evaluation of bone marrow biopsy specimens obtained at the platelet nadir, which demonstrated ample numbers of megakaryoctyes, most of which had actually lost their characteristic appearance, and failed to demonstrate evidence of terminal budding. There were no incidents of neutropenic fever or neutropenic sepsis.

The number of patients with dose-limiting toxicities (DLTs), defined as either grade 3 or 4 non-haematologic toxicities or treatment delay for toxicity for longer than 1 week, for each dose level in the i.v. or oral vorinostat groups are summarised in Table 3. Dehydration, diarrhoea, and fatigue were the predominant DLTs in patients receiving oral vorinostat. The 400 mg daily oral dose was the best tolerated, with only 18% of patients experiencing DLT. In patients receiving i.v. vorinostat, neutropenia or thrombocytopenia as well as dehydration and diarrhoea were the most frequent DLTs.

## ANTITUMOUR ACTIVITY

**Figure 4:**
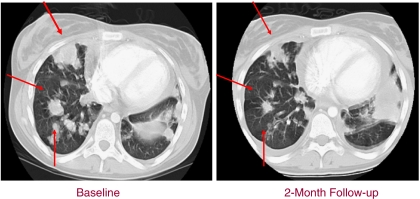
CT scan of thoracic cavity of a patient with refractory HD before and after i.v. vorinostat 600 mg m^−2^ for 5 days every 3 weeks.

Intravenous vorinostat resulted in antitumour activity in three patients with HD who had previously failed autologous transplants (Table 4) (Kelly *et al*, 2003, 2005; O'Connor *et al*, 2006). One HD patient, who was maintained on vorinostat at a dose of 300 mg m^−2^ for 9 months, showed stabilisation of her disease on a computed tomography (CT) scan and normalisation of her positron emission tomography (PET) scan. This patient subsequently converted to oral vorinostat for ease of therapy. The second HD patient receiving i.v. vorinostat at a dose of 600 mg m^−2^ showed a 15% shrinkage of disease, which was associated with a marked improvement in her pulmonary and overall performance status (Figure 4). Her disease subsequently progressed during a period of therapy cessation owing to secondary systemic infection. The third HD patient demonstrated an approximately 42% regression of their disease (durable for 2 months) with symptomatic improvement.

**Figure 5:**
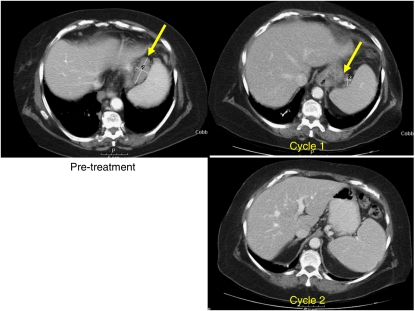
Resolution of a gastrohepatic mass in a patient with transformed DLBCL following oral vorinostat therapy. CT scans were taken before treatment and after one and two cycles of oral vorinostat.

Oral vorinostat also resulted in marked antitumour activity in a total of four patients, one with HD, two with transformed DBLCL, and one with cutaneous T-cell lymphoma (CTCL) (Table 4). The patient with HD showed a 31% tumour response lasting for 9 months, as assessed by CT scan. In the two patients with transformed DBLCL, both of whom were heavily pretreated before receiving vorinostat therapy, one experienced a CR lasting for 12 months and one experienced a PR lasting over 5 months. Figure 5 shows the resolution of a gastrohepatic mass over time in one of the patients with transformed DBLCL who received oral vorinostat. A substantial reduction in tumour size was apparent after one cycle of oral vorinostat therapy, and by two cycles, the mass had completely disappeared. This patient also demonstrated a negative PET scan performed after 5 months of therapy. The patient with CTCL exhibited a PR of his skin tumour over 4 months.

## CONCLUSIONS

The phase I clinical trial experience in patients with haematological malignances showed that both i.v. and oral formulations of vorinostat are well tolerated. The DLTs of i.v. vorinostat were primarily haematologic toxicities, which were rapidly reversible within 4–5 days of therapy cessation. For oral vorinostat, the DLTs were primarily non-haematologic toxicities (dehydration, anorexia, diarrhoea, fatigue), which were also rapidly reversible with a median duration of 3 days. The differences in the types of DLTs observed between oral and i.v. vorinostat may result from dissimilarities in the pharmacokinetics of the two formulations. Intravenous vorinostat administration may give rise to higher *C*_max_ values and lower AUC values than oral vorinostat, and this may have important ramifications in terms of the spectrum of toxicities observed.

Evidence of inhibition of HDAC activity in both PBMNC and tumour tissue biopsies was apparent for up to 10 h following oral administration of vorinostat. Inhibition of HDAC activity was associated with objective tumour regression and symptomatic improvement (with little clinical toxicity) in patients with a variety of haematological malignances, including HD and select subtypes of NHL, including T-cell lymphomas. Further studies are needed to define relevant biologic end points that can be used as a guide to optimise the dosing schedule for vorinostat, particularly with respect to dose, timing of administration, and duration of therapy. At the molecular level, further research is needed to fully delineate the mechanism(s) of antitumour effect of vorinostat in various types of malignances and to understand why normal cells are apparently more resistant to apoptosis than transformed cells. Several phase II studies are currently ongoing in patients with haematological malignances and solid tumours. The preliminary results of vorinostat's activity in T-cell lymphoma will be discussed by Dr Duvic.

## Figures and Tables

**Table 1:** Summary of patient characteristics in i.v. and oral vorinostat groups

	**i.v. vorinostat**	**Oral vorinostat**
Total number of patients	14 Registered	25 Registered
	12 Treated	23 Treated
Male	7 (58%)	16 (69%)
Female	5 (42%)	7 (30%)
Median age	39 (19–77)	57 (20–99)
*Diseases*		
Hodgkin's disease	5 (42%)	7 (30%)
DLBCL	5 (42%)	7 (30%)
Small lymphocytic	1 (8%)	1 (4%)
Mantle cell NHL	—	2 (9%)
CTCL/PTCL	1 (8%)	2 (9%)
Multiple myeloma	—	2 (9%)
APL/MDS	—	1 (4%)/1 (4%)
Prior chemotherapy or biological therapies per patient (median)	7 (4–15)	5 (1–11)
Patients who received prior stem cell transplant	6 (50%)	8 (35%)
Received rituximab	7 (six non-Hodgkin's lymphoma and one Hodgkin's disease)	11 (10 non-Hodgkin's lymphoma and one Hodgkin's disease)

APL=acute promyelocytic leukaemia; CTCL=cutaneous T-cell lymphoma; DLBCL=diffuse large B-cell lymphoma; i.v.=intravenous; MDS=myelodysplastic syndrome; NHL=non-Hodgkin's lymphoma; PTCL=peripheral T-cell lymphoma; SAHA=suberoylanilide hydroxamic acid.

**Table 2:** Common overall non-haematologic and haematological toxicities (grades 3 and 4)

	**i.v. vorinostat (*n*=12)**		**Oral vorinostat (*n*=23)**	
	**Grade 3**	**Grade 4**	**Grade 3**	**Grade 4**
	*Non-haematological toxicities*			
Anorexia	0	0	3	0
Constipation	1	0	0	0
Diarrhoea	1	0	6	0
Dehydration	0	0	11	0
Dyspnea	2	0	0	0
Fatigue	2	0	5	0
Infection w/o neutropenia	1	0	5	0
Hyperglycemia	0	0	3	0
Hypocalcemia	0	0	1	0
Hypokalemia	0	0	2	0
Hypophosphatemia	1	0	4	0
Neurological, Guillan Barre	0	0	1	0
Pleuritic chest pain	1	0	0	0
Thrombosis	1	0	1	0
				
	*Haematological toxicities*			
Anaemia	5	0	4	3
Leukopenia	1	1	3	1
Neutropenia	0	1	3	1
Thrombocytopenia	1	0	7	1

i.v.=intravenous, SAHA=suberoylanilide hydroxamic acid.

**Table 3:** Dose-limiting toxicities (grade 3 or 4 non-haematologic toxicities or treatment delay for toxicity for longer than 1 week) associated with i.v. or oral vorinostat therapy

**i.v. vorinostat**		**Oral vorinostat**	
**Dose level**	**Dose-limiting toxicities**	**Dose level**	**Dose-limiting toxicities**
300 mg m^−2^ day^−1^ × 5 day × 3 weeks (*n*=7)	None	400 mg once daily (*n*=11)	Dehydration/diarrhoea/fatigue (*n*=2)
600 mg m^−2^ day^−1^ × 5 day × 3 weeks (*n*=5)	Treatment delay for neutropenia or thrombocytopenia (*n*=2)	400 mg twice daily (*n*=3)	Dehydration/fatigue/anorexia (*n*=3)
600 mg once daily (*n*=3)	Dehydration/diarrhoea (*n*=2)	600 mg once daily (*n*=3)	Dehydration/fatigue/diarrhoea (*n*=2)
		200 mg twice daily (*n*=6)	Dehydration/anorexia/fatigue (*n*=2)

**Table 4:** Antitumour activity of i.v. and oral vorinostat

**i.v. vorinostat (*n*=12)**		**Oral vorinostat (*n*=23)**	
**Disease**	**Tumour response by CT scan**	**Disease**	**Tumour response by CT scan**
HD	PR × 9 months	tDBLCL	CR × >12 months
HD	SD × 2 months	tDBLCL	PR × >5 months
HD	SD × 3 months	HD	SD × 9 months
		CTCL^*^	PR × 4 months

CR=complete remission; CT=computed tomography; CTCL=cutaneous T-cell lymphoma; DLBCL=diffuse large B-cell lymphoma; HD=Hodgkin's disease; i.v.=intravenous; PR=partial response; SAHA=suberoylanilide hydroxamic acid; SD=stable disease.^*^Response based on skin examination.
